# Human virus and microbial indicator occurrence in public-supply groundwater systems: meta-analysis of 12 international studies

**DOI:** 10.1007/s10040-017-1581-5

**Published:** 2017-04-26

**Authors:** G. Shay Fout, Mark A. Borchardt, Burney A. Kieke, Mohammad R. Karim

**Affiliations:** 1 US Environmental Protection Agency, 26 Martin Luther King Dr, Cincinnati, OH 45268 USA; 2 US Department of Agriculture, 2611 Yellowstone Dr, Marshfield, WI 54449 USA; 3 Marshfield Clinic Research Foundation, 1000 Oak Ave, Marshfield, WI 54449 USA; 4 City of Santa Cruz, Public Works Department, 110 California St, Santa Cruz, CA 95060 USA

**Keywords:** Contamination, Drinking water, Enteric virus, Microbial indicators, Health

## Abstract

Groundwater quality is often evaluated using microbial indicators. This study examines data from 12 international groundwater studies (conducted 1992–2013) of 718 public drinking-water systems located in a range of hydrogeological settings. Focus was on testing the value of indicator organisms for identifying virus-contaminated wells. One or more indicators and viruses were present in 37 and 15% of 2,273 samples and 44 and 27% of 746 wells, respectively. *Escherichia coli* (*E. coli*) and somatic coliphage are 7–9 times more likely to be associated with culturable virus-positive samples when the indicator is present versus when it is absent, while F-specific and somatic coliphages are 8–9 times more likely to be associated with culturable virus-positive wells. However, single indicators are only marginally associated with viruses detected by molecular methods, and all microbial indicators have low sensitivity and positive predictive values for virus occurrence, whether by culturable or molecular assays, i.e., indicators are often absent when viruses are present and the indicators have a high false-positive rate. Wells were divided into three susceptibility subsets based on presence of (1) total coliform bacteria or (2) multiple indicators, or (3) location of wells in karst, fractured bedrock, or gravel/cobble settings. Better associations of some indicators with viruses were observed for (1) and (3). Findings indicate the best indicators are *E. coli* or somatic coliphage, although both indicators may underestimate virus occurrence. Repeat sampling for indicators improves evaluation of the potential for viral contamination in a well.

## Résumé

La qualité des eaux souterraines est. souvent évaluée à partir d’indicateurs microbiens. Cette étude examine des données de 12 études internationales sur les eaux souterraines (réalisées entre 1992 et 2013) concernant 718 systèmes publics d’alimentation en eau potable situés dans une gamme de contextes hydrogéologiques. L’accent a été mis sur l’évaluation de la valeur des organismes indicateurs pour l’identification des puits contaminés par les virus. Un ou plusieurs indicateurs et virus étaient présents respectivement dans 37 et 15% des 2273 échantillons et 44 et 27% des 746 puits. *Escherichia coli* (*E. coli*) et les coliphages somatiques sont 7 à 9 fois plus susceptibles d’être associés à des échantillons mis sous culture positifs pour les virus lorsque l’indicateur est. présent versus quand il est. absent, tandis que les coliphages F-spécifiques et somatiques sont 8 à 9 fois plus susceptibles d’être associés à des puits positifs pour les virus après mises en culture. Cependant, les indicateurs uniques ne sont que marginalement associés aux virus détectés par les méthodes moléculaires, et tous les indicateurs microbiens ont une faible sensibilité et des valeurs prédictives positives concernant la présence du virus, que ce soit par des tests de mise en culture ou des essais moléculaires, par ex., les indicateurs sont souvent absents lorsque les virus sont présents et les indicateurs ont un taux de faux positif élevé. Les puits ont été divisés en trois sous-ensembles de susceptibilité basés sur la présence de (1) bactéries coliformes totales, ou (2) d’indicateurs multiples, ou (3) la localisation des puits dans des contextes karstiques, de socle fracturé, ou de graviers/galets. De meilleures associations de certains indicateurs avec virus ont été observées pour (1) et (3). Les résultats indiquent que les meilleurs indicateurs sont *E. coli* ou le coliphage somatique, bien que les deux indicateurs peuvent sous-estimer la présence des virus. L’échantillonnage répété pour identifier les indicateurs améliore l’évaluation du potentiel de contamination virale dans un puits.

## Resumen

La calidad del agua subterránea se evalúa a menudo utilizando indicadores microbianos. Este estudio examina los datos de 12 estudios internacionales de aguas subterráneas (llevadas a cabo entre 1992–2013) de 718 sistemas públicos de agua potable localizados en una gama de escenarios hidrogeológicos. El objetivo fue probar el valor de los organismos indicadores para identificar los pozos contaminados por virus. Uno o más indicadores y virus estuvieron presentes en 37 y 15% de las 2273 muestras y 44 y 27% de 746 pozos, respectivamente. *Escherichia coli* (*E. coli*) y colifagos somáticos son 7–9 veces más probables de asociarse con muestras cultivables positivas al virus cuando el indicador está presente frente a cuando está ausente, mientras que los colifagos F específicos y somáticos están 8–9 veces más probablemente asociados con pozos cultivables positivos para el virus. Sin embargo, los indicadores individuales sólo están ligeramente asociados con los virus detectados por métodos moleculares, y todos los indicadores microbianos tienen baja sensibilidad y valores predictivos positivos para la ocurrencia del virus, ya sea por ensayos cultivables o moleculares, es decir, los indicadores están a menudo ausentes cuando los virus están presentes y los indicadores tienen una alta tasa de falsos positivos. Los pozos se dividieron en tres subconjuntos de susceptibilidad basados en la presencia de (1) bacterias coliformes totales, o (2) indicadores múltiples, o (3) localización de pozos en rocas kársticas, roca fracturada o configuración de grava/canto rodado. Se observaron mejores asociaciones de algunos indicadores con virus para (1) y (3). Los hallazgos indican que los mejores indicadores son *E. coli* o colifagos somáticos, aunque ambos indicadores pueden subestimar la ocurrencia del virus. La repetición del muestreo para los indicadores mejora la evaluación del potencial de contaminación viral en un pozo.

## 摘要

常常采用微生物指标评估地下水水质。本研究梳理了不同水文地质背景下718个饮用水供水系统其中(1992–2013年进行的)12项国际研究的资料。重点测试指标微生物值以确定病毒污染井。在2,273个样品中及746口井中,一种或更多种指标和病毒分别为37和15%以及44%和27%。存在着指标与指标缺失相比,很可能与可培养的病毒阳性样品相关的埃希氏杆菌属大肠杆菌及体细胞大肠杆菌噬菌体为7–9倍,而很可能与可培养的病毒-阳性井相关的F-特定的、体细胞大肠杆菌噬菌体为8–9倍。然而,单个指标与通过分子法检测出的病毒关系不大,所有微生物指标对病毒的出现具有很低的灵敏度及很低的阳性预测值,无论是通过可培养的或是通过分子实验,也就是说存在病毒时,指标常常缺失,并且指标的假阳性率很高。根据1)总大肠菌细菌存在情况或2)多种指标存在情况或3)岩溶断裂基岩或砾石/鹅卵石环境下井的位置,井被分为三个敏感带子项。对于1)和3),观测到有些指标与病毒关联性较好。这些发现表明,最好的指标是埃希氏杆菌属大肠杆菌及体细胞大肠杆菌噬菌体,尽管这两种指标可能会低估病毒的发生。指标的重复采样提高了井中病毒性污染潜力的评估水平。.

## Resumo

A qualidade das águas subterrâneas é muitas vezes avaliada usando indicadores microbianos. Este estudo examina 12 estudos internacionais (conduzidos entre 1992-2013) sobre 718 sistemas públicos de água potável, localizados em uma grande faixa de tipos de regiões hidrogeológicas. O foco foi em testar o valor de organismos indicadores para identificar poços contaminados por vírus. Um ou mais indicador e vírus estavam presentes em 37 % e 15 % das 2,273 amostras e 44 % e 27 % dos 746 poços, respectivamente. *Escherichia coli* (*E. coli*) e colifagos somáticos são 7–9 vezes mais prováveis de serem associados com amostras cultivadas positivas para vírus quando o indicador está presente versus quando está ausente, enquanto F-específicos e colifagos somáticos são 8–9 vezes mais prováveis de estarem associados com poços com culturas positivas para vírus. Entretanto, indicadores individuais são apenas associados marginalmente com vírus detectáveis por métodos moleculares, e todos os indicadores microbianos têm baixa sensitividade e valores preditivos positivos para ocorrência de vírus, tanto para testes de cultura ou moleculares, p. ex., os indicadores são muitas vezes ausentes quando vírus estão presentes e os indicadores têm uma alta taxa de falsos positivos. Os poços foram divididos em três subconjuntos de suscetibilidade baseados na presença de (1) bactérias coliformes totais, ou (2) vários indicadores, ou (3) localização de poços em rochas fraturadas e cársticas, ou ambientes com cascalho/pedregulho. Associações melhores de alguns indicadores com vírus foram observados em (1) e (3). Os resultados indicam que os melhores indicadores são *E. coli* ou colifagos somáticos, apesar que ambos os indicadores podem subestimar a ocorrência de vírus. Amostragens repetidas dos indicadores melhoram a avaliação de possível contaminação por vírus em um poço.

## Introduction

Groundwater is an important source of drinking water in both developed and developing countries. It constitutes about 95% of the world’s accessible freshwater (Chilton and Seiler 2006; Howard et al. 2006; McKay 2011) and is often used with little or no treatment (Pedley et al. 2006). Contamination of groundwater with human enteric viruses is a global issue (Blaschke et al. 2016; Gotkowitz et al. 2016; Hynds et al. 2014; USEPA 2006a, b), as consumption of contaminated water can result in elevated rates of endemic illness and waterborne disease outbreaks in affected communities (Beer et al. 2015; Borchardt et al. 2011; Cho et al. 2014; Guzman-Herrador et al. 2015; Hilborn et al. 2013; Jack et al. 2013; Wallender et al. 2014; Zhou et al. 2012). Enteric viruses implicated in waterborne outbreaks include enteroviruses, hepatitis A, rotavirus, and norovirus (Craun et al. 2010; Hejkal et al. 1982), but others such as adenoviruses, Aichi virus 1, hepatitis E, and reoviruses, potentially are capable of groundwater-borne transmission. Waterborne viruses cause a wide range of illnesses, including gastroenteritis, paralysis, aseptic meningitis, conjunctivitis, diabetes, fevers, herpangina, rash, myocarditis, and respiratory illness, (Kitajima and Gerba 2015; WHO 2011).

Several nonpathogenic bacteria that are normal flora of the human intestine and other warm-blooded animals can be detected using simple and inexpensive techniques. These include total coliform bacteria, fecal (thermotolerant) coliform bacteria, *E. coli*, enterococci, and bacterial endospores (Ashbolt et al. 2001; Locas et al. 2007; Tallon et al. 2005). Because pathogens, and especially viral pathogens, occur sporadically and are difficult to detect, these bacteria have been used as indicators of fecal pollution. Total coliform bacteria and *E. coli* are the most commonly employed water quality indicators, but of the two, *E. coli* is a more definitive indicator of fecal pollution (Edberg et al. 2000; Tallon et al. 2005).

Coliphages (bacteriophages that infect coliform bacteria) are present in wastewater and have been suggested to be a useful conservative indicator of fecal and viral pollution of groundwater (Deborde et al. 1998a, b; Lucena et al. 2006). They are grouped into two major categories—F-specific and somatic coliphages. F-specific coliphages are similar in size (about 26 nm in diameter) and structure (icosahedral) to human enteric viruses (about 28–38 nm in diameter for enteroviruses and noroviruses), while somatic coliphages have more variability. Unlike enteric viruses, coliphages are detectable by simple, inexpensive and rapid techniques (Lucena et al. 2006). Phages of *Bacteroides* have also been evaluated as useful indicators (Jofre et al. 1986; Johnson et al. 2011; McMinn et al. 2014).

Although bacterial indicators are used for general water quality monitoring, they are not considered to be adequate indicators of viral contamination because the structure, composition, morphology, size (about 1,000 nm in diameter × 2,000 nm in length for *E. coli*), and survival characteristics of viruses differ fundamentally from those of bacteria (Ashbolt 2015; Payment and Locas 2011; WHO 2011). Viruses survive longer than vegetative bacteria in the environment (Nasser et al. 1993) and have different transport properties. The usefulness of anaerobic bacterial endospores is limited by very long survival times, the ubiquity of *Clostridium* species in soil, and by transport properties (Meschke 2001). Aerobic endospores also have major differences in transport and survival properties (Headd and Bradford 2016; Pang et al. 2005).

Viral and other pathogens and microbial indicators enter aquifers through multiple sources and pathways, including leachates from sanitary landfills, on-site septic waste treatment discharges, broken sewage lines, runoff from urban, agricultural and natural areas, and water reuse by direct injection of inadequately treated wastewater into aquifers (Borchardt et al. 2012; Costan-Longares et al. 2008; Gotkowitz et al. 2016). Fecal contamination from the surface may also get into groundwater through improperly constructed, protected, or maintained wells (Hynds et al. 2014).

Despite the potential public health impact from drinking untreated groundwater, the regulations guiding the quality of groundwater supplies used for drinking water are highly varied among countries. Many use the World Health Organization Guidelines for Drinking Water Quality (WHO 2011), which covers drinking water from all sources and suggests that drinking water contain no thermotolerant coliform bacteria or *E. coli* per 100 ml. Canada’s guidelines state that drinking water should have no detectable total coliforms or *E. coli* per 100 ml (Health Canada 2017). Australia’s guidelines add intestinal enterococci and coliphages in addition to thermotolerant bacteria and *E. coli* as agents that should not be detected in 100 ml and suggest that sanitary surveys be conducted for groundwater systems (National Health and Medical Research Council 2016). Sanitary surveys are used to examine a system’s deficiencies that could cause vulnerability to microbial contamination. The surveys are intended to identify deficiencies caused by poor source water, inadequate well construction or maintenance, and improper system operation. Monitoring of water quality is required by regulation in some countries—for example, Japan requires monitoring for *E. coli* (Wakayama 2016). Korea does periodic monitoring for enteric viruses and their Groundwater Act requires monitoring of groundwater for *E. coli* (Lee and Kwon 2016; Lee H et al. 2011; Lee S et al. 2011). The European Union requires regulatory authorities to monitor all public drinking-water systems for coliform bacteria and *E. coli* at a frequency that depends on water volume pumped and/or population served. The United States regulatory instrument is the Ground Water Rule (GWR; USEPA 2006a, b). The key components of the GWR are: sanitary surveys, triggered source water monitoring, corrective actions, and compliance monitoring. Source monitoring may be required rather than triggered if the source water quality is uncertain. The triggered water monitoring provision requires that untreated groundwater systems must conduct triggered source water monitoring for the presence of at least one of the three fecal indicators: *E. coli*, enterococci, or coliphage following a Revised Total Coliform Rule (RTCR)-positive sample. The RTCR requires regular monitoring of all public systems for total coliforms using 100-ml samples at a frequency that depends on the population served. Any regular monitoring sample that contains any amount of total coliforms must be followed by additional tests for both total coliforms and *E. coli*. The compliance monitoring provision of the GWR requires that systems that provide 4-log treatment of viruses must conduct compliance monitoring to demonstrate continual treatment effectiveness. The GWR uses a risk-targeted approach to address the likelihood of viral contamination of wells—i.e., well susceptibility to contamination (note: susceptibility herein is defined by the entry of fecal contamination into an aquifer as measured by the demonstrated presence of virus in any sample from an associated well). It suggests that the agencies that implement the rule consider groundwater from aquifers in limestone, igneous and metamorphic rock, and gravel as potentially susceptible. However, among aquifers in gravel settings, gravel/cobble aquifers are more susceptible than those consisting of gravel/sand (Berger 2008). Aquifers in gravel settings have rock grain sizes of 4 mm or larger, whereas those in gravel/cobble settings have grain sizes up to about 256 mm (Wentworth 1922).

While a good indicator would be expected to be positively correlated to virus presence (i.e., always present when viruses are present and absent when viruses are absent), these types of correlations are not always observed in groundwater (Payment and Locas 2011). This might be due to indicator and virus differing in die-off properties, transport characteristics, waste treatment practices before release to the environment, source concentrations—e.g., indicators are constantly present and at higher concentrations in wastewater than are viruses (Berg et al. 1978)—and virus infection dynamics and shedding rates in the host population. In addition, in an analysis of studies correlating indicators and pathogens in water types other than groundwater, Wu et al. (2011) showed that the strength of correlations was related to study sample size and the number of pathogen-positive detections. Mindful of these limitations, this study examined the relationship between indicators and viruses by combining data from 12 studies of public drinking-water groundwater systems. In addition to conventional tests of correlation, the study evaluated the association between indicator and virus occurrence by logistic regression and calculated standard performance measures for diagnostic tests, namely, sensitivity, specificity, positive predictive value, and negative predictive value. These measures were further evaluated for three subsets of wells considered susceptible to fecal contamination based on hydrogeological setting and US regulations.

## Materials and methods

### Site selection

Raw data from 12 studies on viruses in groundwater were used to determine the association between microbial indicators and virus occurrence. Groundwater studies were selected primarily on the basis of availability of raw data supplied in publications or from the corresponding authors (Table 1). Secondly, studies were selected to give a broad range of hydrogeological settings in North America, Europe, and Asia, and to avoid over-representing one location or study team in the combined dataset.

**Table 1:** Microbial indicator and virus occurrence sources used in this study^a^

Study name	Number of drinking water systems	Number of wells	Number of samples	Study date range (month/year)	Reference
USEPA/AWWARF	29	30	333	9/92–12/94	Dahling 2002; Fout et al. 2003; Lieberman et al. 2002
USGS (Missouri)	180	182	322	5/97–7/98	Davis and Witt 2000; Femmer 2000
USGS (Ohio)/USEPA	38	38	169	6/99–7/01	Francy et al. 2004
USGS (Pennsylvania)	60	60	60	9/00–2/01	Lindsey et al. 2002
American Water Service Company	20	20	235	3/01–6/02	Karim et al. 2004
University of Tennessee (USA)	4	4	6	3/04–8/04	Johnson et al. 2011
Institut Armand-Frappier (Canada)	36	36	243	3/04–12/12	Locas et al. 2007; Locas et al. 2008; Payment and Locas 2005; P. Payment, Institut Armand-Frappier, 2015, personal communication
University of Rome Tor Vergata (Italy)	8	8	14	6/05–12/05	Gabrieli et al. 2009
University of Tokyo (Tokyo, Japan)	46	46	46	11/05–1/06	Katayama 2008; H. Katayama, University of Tokyo, 2015, personal communication
Marshfield Clinic Research Foundation (USA)	14	36	391	4/06–11/07	Borchardt et al. 2012
NIER (South Korea)	220	220	383	7/07–12/08	Jung et al. 2011; Lee et al. 2013, ; Lee H et al. 2011; Lee S et al. 2011; S-Y. Paik, The Catholic University, Seoul, 2015, personal communication
Iowa Department of Natural Resources (USA)	63	66	71	3/13–6/13	Hruby et al. 2015
Total	718	746	2,273	–	–

^a^All wells were from public drinking water systems, e.g., those used by multiple individuals rather than a single household. In the US, a public system is one with at least 15 service connections or which serves 25 persons for 60 days or more

### Summary of included studies

A summary of each study is provided with a focus on settings and virus occurrence. The American Water Works Association Research Foundation (AWWARF, now Water Research Foundation) and USEPA conducted a study of viruses in groundwater in the US public systems during 1992–1994 (Dahling 2002; Fout et al. 2003; Lieberman et al. 2002; USEPA 2006a, b). This study focused on 30 wells, with 13 located in karst, fractured bedrock, or course gravel settings; 11 in alluvial settings with frequent microbial indicator-positive samples, and 6 in unknown or in alluvial settings without a record of frequent indicator detections. All but one of the seven culturable virus-positive wells were in karst or fractured bedrock locations, while only 11 of the 25 sites positive for virus by molecular tests were in karst or fractured bedrock settings. A culturable virus-positive sample means that infectious viruses are present in water from the aquifer. Culturable virus methods detect only a narrow range of those viruses that cause waterborne disease, so a negative result should not be interpreted to mean that all infectious viruses are absent. Molecular methods can detect most of the viruses that cause waterborne illness, but most studies only assay for a limited number of them. The primary limitation of molecular methods is their inability to determine whether detected viruses are infectious. They also may be affected by water chemistries that cause a false-negative reaction.

The US Geological Survey (USGS) and Public Drinking Water Program of the Missouri Department of Natural Resources conducted a two-phase study of 182 public water supplies in the Ozark plateaus aquifer system in Missouri during 1997–1998 (Davis and Witt 2000; Davis and Witt 1998; Femmer 2000). Public supply wells were selected to represent settings in primary karst, secondary karst, the confined Ozark aquifer, or in alluvium. One sample was positive for culturable virus and that was from a source located in an area of confined primary karst; however, only three of the 13 samples positive by molecular assays were in karst settings.

Francy et al. (2004a, b) conducted a study of 38 groundwater-supply wells during 1999–2001. This study targeted public systems that served a population size between 25 and 3,300 in Silurian-Devonian sand, gravel, and clay aquifers in southeastern Michigan, USA. The aquifers in this area consist typically of dual layers of glacial outwash and till with unconfined upper layers and semi-confined lower layers. Culturable virus was detected at two of these sites and virus by molecular tests at seven.

The USGS conducted a study of microbial indicators and virus from 60 non-community public water supplies in Pennsylvania during 2000–2001 (Lindsey et al. 2002). Twenty-five sites were located in karstic limestone or dolomite, with another 25 sites situated in fractured bedrock. Five sites each were in siliciclastic settings and in unconsolidated sediments. Culturable viruses were detected in two wells in a karstic setting, at one well in a fractured bedrock setting, and at two wells in a siliciclastic setting.

The American Water Service Company performed a study of 20 public drinking water wells from 11 US states during 2001–2002. Wells were selected from the first round of a larger study (Abbaszadegan et al. 2003) based upon the presence of culturable virus (five wells), viral nucleic acid (six wells), indicator bacteria (five wells), and an absence of both indicators and virus (four wells). Three of the wells were in fractured bedrock settings, with most others being in alluvial or glacial sand and gravel. Culturable virus was present at seven sites and virus by molecular assays at 15 sites; two of the three wells in fractured bedrock were virus-positive, one for culturable virus and the other for virus measured by PCR.

A small study of springs and wells was conducted in Tennessee (USA) during 2004 (Johnson et al. 2011). Three wells were selected from a larger group of wells to represent “low” susceptibility to fecal contamination. This was based upon the absence of *E. coli* during an initial period of monitoring of the wells for microbial indicators (Johnson 2005). One of these wells was in a Pre-Conasauga group carbonate aquifer overlain by 35 m of residuum. Another was in a Copper Ridge dolomite (Knox Group) aquifer, overlain by 30–46 m of overburden. The third was in Chilhowee Group Sandstone and Conglomerate aquifer, overlain by 0–2 m of residuum. The fourth well, located in a Knox Group Carbonate aquifer, overlain by 9 m of residuum, was chosen to represent a susceptible well based upon the prior sampling. Culturable virus was detected at two of the three wells in the low susceptibility group and in the well in the high susceptibility group.

A year-long study of 12 municipal wells in Quebec, Canada, was conducted during 2003–2004 to examine the influence of different aquifers, soil types, and well depths on virus and indicator occurrence (Locas et al. 2007). Groups of public systems were selected consisting of (1) wells tapped into glacial deposits with and without confinement and no history of microbial detections; (2) wells in glacial deposits or fractured bedrock with sporadic detection of total coliforms; and (3) wells in unconfined sand and gravel aquifers or fractured sandstone with frequent detections of total and fecal indicators. A follow-up study was conducted during 2006 and 2012 on 24 municipal wells in three provinces in Canada (Locas et al. 2008; Payment and Locas 2005; P. Payment, Institut Armand-Frappier, 2015, personal communication), including two group three sites from the first study. No data were collected on the hydrogeology of the additional wells, but none appeared to be in karst or fractured bedrock locations (Ford 1997). Overall, culturable virus was detected at four sites, two of which were in karst or fractured bedrock sites. Norovirus was detected at three unconfined aquifers.

A small study of eight groundwater wells in karstic settings was conducted during 2005 in central Italy by the University of Rome Tor Vergata (Gabrieli et al. 2009). Samples were tested for virus using only molecular assays. All were negative for fecal indicators, while three wells (38%) were positive for norovirus.

A Japanese study of 46 wells was conducted by the University of Tokyo during 2005–2006 in the eastern lowland area of Tokyo (Katayama 2008). About half of the wells received water from the unconfined Yurakucho alluvial sand aquifer, while the other half obtained water from deeper confined aquifers. Samples were tested for adenovirus using real time PCR with four wells being positive, two of which were in confined and two in unconfined aquifers.

A study of 36 public wells that supplied untreated drinking water to 14 small communities (population of 1,363–8,300) was conducted by the Marshfield Clinic Research Foundation in Wisconsin (USA) during 2006–2007 (Borchardt et al. 2012). Wells were primarily located in non-karstic, sandstone settings (Lambertini et al. 2011). The setting of six communities was sand and gravel or mixtures of sand, gravel, and sandstone. Two communities had limestone and dolomite or sandstone with limestone and dolomite aquifers. Two communities were located close to regions in the state that are karstic, but do not appear to be influenced by karst. Well depths ranged from 19 to 173 m and pumping rates from about 500,000 L/day for the smallest community to 14,500,000 L/ day for the largest community. All samples were tested for adenovirus, enterovirus, hepatitis A virus, norovirus genogroups I and II, and rotavirus by real time PCR. Samples that were positive for adenovirus and enterovirus by PCR were also tested for culturable viruses using integrated cell culture-quantitative PCR. Overall, 31 of the wells were positive for virus and about a quarter of the PCR-positive samples tested contained culturable virus.

South Korea’s National Institute of Environmental Research (NIER) initiated several groundwater monitoring studies in metropolitan areas and provinces during 2007–2011 (Jung et al. 2011; Lee et al. 2013; Lee H et al. 2011; Lee S et al. 2011) with data being provided for 220 sites. No information was given on hydrogeology of the sites, but four were in areas with porous volcanic rock or with a high likelihood of being in karst regions (University of Auckland 2008). Other sites would be primarily alluvial as the hydrogeology of most of the Korean peninsula is poorly permeable crystalline granitic and metamorphic rocks (Won et al. 2005), and thus associated with low groundwater yields. Wells were frequently positive for viruses by PCR, with 30% being positive for norovirus, 13% for adenovirus, and 8% for enterovirus. No virus was present in the volcanic or karst sites. More than half of the norovirus positive samples were from genogroup I genotypes.

A study of public systems in Iowa (USA) was conducted in the spring of 2013 (Hruby et al. 2015). A total of 63 systems (with 66 wells) were chosen covering the major hydrogeological areas of the state. These included aquifers in alluvial settings (18% of wells), sand and gravel (11%), Cambrian-Ordovician sandstone (18%), Dakota Cretaceous sandstone (11%), Mississippi sandstone and carbonate (8%), and Silurian-Devonian carbonate (35%). Although a few areas in the northeast portion of the state have strong karst features (Horick 1984), all of the studied wells appear to be outside the karst areas. Samples were analyzed for microbial indicators, pathogens, and numerous chemical contaminants. Despite the large number of wells in carbonate and sandstone aquifers, there was virtually no microbial contamination of the wells. One site had norovirus genogroup II, but all were negative for adenoviruses, enteroviruses, or hepatitis E virus. Samples were collected following a severe drought during 2012, which may have been a contributing factor to the low microbial contamination observed during the study. This study was the first to include measurements of pepper mild mottle virus (PMMV). This virus comes from foods eaten with peppers and occurs at high levels in sewage (Kuroda et al. 2015). Eleven samples in the Iowa study were positive for PMMV, suggesting that this virus may be a better conservative indicator of microbial contamination from human sources than bacterial or bacteriophage indicators; however, Kuroda et al. (2015) suggest the detection rate for PMMV in groundwater is lower than that of human enteric viruses.

### Data handling

This study analyzed available raw data from the 12 groundwater virus occurrence studies described in the preceding, covering 2,273 samples from 718 drinking water systems and 746 wells (Table 1). Table 2 describes the quantity and type of data provided by each study. Culturable virus occurrence and virus occurrence measured by PCR methods (hereafter designated PCR-virus) was provided by six studies. One study provided only culturable virus occurrence data, while five studies provided only PCR-virus data. Some studies provided qualitative data for some analytes. For purposes of analysis, all qualitative data were converted to quantitative data as detailed in Tables 3 and 4. In addition, all concentrations were normalized to 100 ml for making comparisons across the studies.

**Table 2:** Assays and data types for combined studies

Study name	TC^a^	EC	Ent	Spores	F+	SomPh	CulVir	PCRVir	Type
USEPA/AWWARF	QN	QN	QN	QN^b^	QN	QN	QN	QL	Yes
USGS (Missouri)	QN^c^	QN	QN	ND	QN	QN	QN	QL^d^	Yes^d^
USGS (Ohio)/USEPA	QN	QN	QN	ND	QN	QN	QL	QL	Yes
USGS (Pennsylvania)	QN	QN	QN	ND	QN	QN	QN	ND	ND
American Water Service Company	QL	QL	QL	QN^b^	QN	QN	QN	QL	Yes
University of Tennessee (USA)	QN	QN	ND	ND	ND	ND	QN	QL	Yes
Institut Armand-Frappier (Canada)	QN	QN	QN	QN^e^	QN	QN	QN	QL	Yes
University of Rome Tor Vergata (Italy)	QN	QN	QN	ND	ND	ND	ND	QL	Yes
University of Tokyo (Tokyo, Japan)	QN	QN	ND	ND	ND	ND	ND	QL	Yes
Marshfield Clinic Research Foundation (USA)	QL^f^	QL^f^	ND	ND	ND	ND	ND	QN	Yes
NIER (South Korea)	QL	QL	ND	ND	QN	QN	ND	QL	Yes
Iowa Department of Natural Resources (USA)	QN	QN	QN	ND	QN	QN	ND	QN	Yes

^a^Abbreviations: *TC* total coliforms; *EC* *E. coli*; *Ent* enterococci; *F+* F-specific phage; *SomPh* somatic phage; *CulVir* culturable virus; *PCRVir* PCR-virus; *Type* individual virus types identified by PCR; *QN* quantitative assays; *QL* qualitative assays (presence/absence); *ND* assay not performed or data not available

^b^Anaerobic spores measured

^c^Fecal rather than total coliforms measured

^d^This study was performed in two phases with no molecular assays being done in the 2nd phase

^e^Aerobic spores measured

^f^Community TCR data were used to estimate the possible presence of total coliform and *E. coli* in community wells of this study. All wells in a community were assumed to be negative if a TCR sample collected within 0-2 days of the day of virus sampling was negative. Likewise, wells were considered positive if a TCR sample was positive during the same timeframe (i.e. 0-2 days). Wells were considered negative for *E. coli* if a total coliform test was negative and *E. coli* data not recorded

**Table 3:** Microbial indicator and culturable virus data conversion ^a^

Indicator/virus	No. volume options ^b^	Volume analyzed	Result	Value/100 ml Used
Bacterial indicators	1	100 ml	-	0 CFU
	1	100 ml	+	5 CFU
	1	100 ml	TMTC ^c^	200 CFU
	2	100 ml	-	0 CFU
		1000 ml	-	
	2	100 ml	-	0.5 CFU
		1000 ml	+	
	2	100 ml	+	5 CFU
		1000 ml	**+**	
Bacteriophage	1	100 ml	TMTC ^c^	400 PFU
	2	100 ml	-	0 PFU
		1000 ml	-	
	2	100 ml	-	0.5 PFU
		1000 ml	**+**	
	2	100 ml	+ or ND ^d^	5 PFU
		1000 ml	+	
Culturable virus	1	500 L	-	0 MPN
1. (expressed as +/- per 500 L	1	500 L	+	0.0002 MPN

^a^Qualitative data were converted to quantitative data as indicated so that Spearman correlations could be performed on the broader data set. The converted data were not used for any other statistical analysis.

^b^No. volume options = 1: a 100-ml sample volume was used for microbial indicator tests; No. volume options = 2: both 100-ml and 1,000-ml sample volumes were used.

^c^Quantitative data using 100-ml samples with too many colonies (bacteria) or plaques (bacteriophage) to count

^d^ND – not done

**Table 4:** Molecular method qualitative data conversion

Assay type	Conversion
Integrated cell culture-PCR	Assuming at least one positive cell culture flask (i.e., 1 MPN per volume assayed), MPN/L is calculated by dividing 1 MPN by the equivalent volume of original water sample placed on replicate flasks. Genomic copies (GC)/100 ml is calculated by dividing the MPN/L value by 10 and multiplying by 20. Note: the value 20 is a conservative estimate of the physical to infectious particle ratio
Conventional PCR	Study-specific data were used to calculate the volume (in liters) of groundwater sample added to each PCR assay. This volume was calculated using the volume of groundwater from which virus was initially concentrated, the volumes and amount of any additional concentration steps, and the volume of final concentrate or extracted nucleic acid added to each PCR assay. GC/100 ml is calculated by dividing 1 by the volume per PCR assay (note that included in this formula is an assumption that the detection limit for PCR is 10 GC. The actual detection limit can vary depending on the presence of PCR inhibitors in a sample)

### Statistical analysis

Spearman rank order correlations (SigmaPlot) between concentrations of viruses and indicators were evaluated at two levels, sample and well. By well, the sum of virus concentrations was compared to the sum of indicator concentrations. As many samples were negative for virus or indicator (i.e., below the detection limit of the assays) which might result in spurious correlations, the analyses were repeated using a dataset restricted to culturable virus and PCR-virus positive samples.

The utility of water quality indicators as indicators of virus-contaminated groundwater was evaluated by testing the association between indicators and virus and by calculating the four conventional performance measures of a diagnostic test: sensitivity (i.e. true positive rate), specificity (i.e., true negative rate), positive predictive value, and negative predictive value (Borchardt et al. 2003) for the 12-study combined dataset. For these association and performance analyses, each of the six indicators—total coliforms, *E. coli*, enterococci, F-specific phage, somatic phage, spores (anaerobic spores + aerobic spores)—was treated as a dichotomous variable (detect or non-detect) and compared to two measures of virus contamination: culturable virus and PCR-virus. Both outcome measures were also treated as dichotomous variables (i.e., detect/non-detect). The strength of the indicator-virus association additionally was quantified by calculating the risk ratio (positive predictive value/[1-negative predictive value]). The null value for the risk ratio is 1.0 with values greater than 1.0 representing the relative elevation in the virus detection rate if the indicator is detected versus when the indicator is not detected. The preceding analyses were conducted separately for data at the level of the sample and well and all results were derived from logistic regression models.

Two logistic regression model formulations that addressed the non-independent nature of the data were employed. The primary analyses entailed fitting mixed models with random intercepts for study and well (sample-level analyses) and study only (well-level analyses). These models also incorporated robust variance estimation (Chavance and Escolano 2016; Morel et al. 2003). The second model formulation involved fitting population-averaged models with robust variance estimation (Morel et al. 2003). With respect to the point estimates of the risk ratio, sensitivity, specificity, positive predictive value and negative predictive value; the mixed model formulation can be considered adjusted (i.e., for study in all analyses and additionally for well in the sample-level analyses), whereas the latter formulation can be viewed as unadjusted. To facilitate model convergence, the independence covariance structure was used in all models. All logistic regression modeling was performed using PROC GLIMMIX of the SAS software (SAS Institute, Inc., Cary, NC).

The aforementioned analytic framework was applied separately to all wells and to three separate non-mutually-exclusive subsets of wells satisfying each susceptibility criterion (Table 5). The first susceptibility subset included all wells that either have or have potential to have multiple violations of the RTCR (hereafter, TCR). The second susceptibility subset included all wells located in karst, fractured bedrock, or gravel/cobble hydrogeological settings. The last subset was based on the triggered source water monitoring provision of the GWR. This included wells with total coliforms and any of the additional follow-up indicators specified by the GWR.

**Table 5:** Susceptibility categories for evaluating microbial indicators

Susceptibility subset	Description
Total Coliform Rule (TCR)	This subset includes all wells from identifiable US groundwater systems (380 of 412 systems) with more than two health-based TCR violations (from EPA’s Safe Drinking Water Information System Fed Data Warehouse database) and all wells from unidentifiable US and international systems that likely would have had violations based on TCR criteria. Two violations were allowed due to the possibility of violations being due to distribution issues rather than from groundwater (Lambertini et al. 2012)
Hydrogeology	This subset includes all wells located in karst, fractured bedrock, or gravel/cobble settings. Many of the individual studies provided information on the hydrogeology of well settings. For those that did not report this information, karst maps were used to determine the setting (University of Auckland 2008; Weary and Doctor 2014)
US Ground Water Rule indicators (GWR)	This subset includes all wells with total coliforms and either *E. coli*, enterococci, or coliphage in any sample from a well (e.g., all US and international wells that might be triggered into additional monitoring based on GWR criteria)

When conducting well-level analyses, an attempt was made to include an adjustment in the regression models for the number of times the well was sampled (NTWS), since this could affect the corresponding probability of detection for the well; however, this resulted in model convergence problems. Within each study NTWS was generally homogeneous, either a small number of similar values or dominated by a single value. It is possible that the model adjustment for study lessened any effect of NTWS, but this may be a limitation of the well-level analyses.

## Results

Table 6 shows the percent of samples and wells that were positive for indicators and virus from the 12 groundwater studies. Total coliforms were detected in 31% of samples and 36% of wells. Overall, 37% of samples and 45% of wells were positive for any indicator, while 15% of samples and 27% of wells were positive for any enteric viruses. The average titer of positive samples containing culturable- or PCR-virus from the 12 studies was 0.4 infectious units and 16 genomic copies per liter, respectively (data not shown).

**Table 6:** Indicator and virus occurrence in groundwater from 12 groundwater virus studies

Target	Percent positive (*n*)		Percent of studies analyzing for the target
	Samples	Wells	
Total coliform	30.7 (2,013)	36.2 (741)	100
*E. coli*	10.5 (2,009)	12.0 (741)	100
Enterococci	12.1 (1,389)	14.9 (424)	67
Aerobic spores	43.1 (188)	52.0 (25)	8
Anaerobic spores	6.0 (567)	26.0 (50)	17
F-specific phage	7.5 (1,799)	15.2 (644)	67
Somatic phage	9.6 (1,801)	11.0 (644)	67
Any indicator	36.9 (2,015)	44.5 (741)	100
Culturable virus	3.9 (1,292)	7.9 (365)	58
PCR-virus	14.1 (2,106)	29.6 (611)	92
Any virus	14.7 (2,273)	26.7 (745)	100
Adenovirus	7.7 (781)	18.3 (191)	33
Enterovirus	5.9 (1,426)	13.6 (413)	67
Norovirus	7.0 (1,597)	21.3 (442)	67
Hepatitis A virus	1.1 (1,072)	9.9 (121)	33
Rotavirus	1.4 (927)	7.8 (115)	33
Reovirus	12.9 (303)	33.3 (60)	17

Spearman Rank Order tests showed that among the indicators there were positive and moderately strong correlations (rho ≥0.5, *P* < 0.001, *n* > 1,300) for combinations of total coliforms, *E. coli*, and enterococci, and between enterococci and somatic phage (data not shown). Between the indicators and culturable virus concentrations on a per-sample basis the trends were positive but weak (rho ≤0.3, *P* < 0.001, *n* > 1,200; Table 7). Correlations on a per-well basis were also weak with the highest between somatic coliphage and culturable virus (rho = 0.46, *P* < 0.001, *n* = 355). Correlations among indicators and PCR-virus (Table 7) were always weaker than to culturable virus.

**Table 7:** Spearman rank order correlation (rho value) of microbial indicators and viruses from the 12 groundwater virus studies^a^

Indicator/virus	Sample-specific		Well-specific	
	Culturable virus	PCR-virus	Culturable virus	PCR-virus
Total coliform	0.22 (1,289)***	0.07 (1,844)**	0.37 (366)***	0.26 (608)***
*E. coli*	0.30 (1,285)***	0.02 (1,840)	0.31 (366)***	0.20 (608)***
Enterococci	0.27 (1,228)***	0.04 (1,222)	0.33 (349)***	0.31 (292)***
Spores	0.17 (755)***	−0.09 (755)**	0.18 (73)	0.00 (73)
F-specific phage	0.22 (1,274)***	0.08 (1,632)**	0.38 (355)***	0.16 (510)***
Somatic phage	0.31 (1,276)***	0.12 (1,634)***	0.46 (355)***	0.29 (510)***
Culturable virus	–	0.13 (1,125)***	–	0.19 (231)**

^a^Correlations were performed in SigmaPlot; number of samples or wells shown *in parentheses*;*P =0.01 to 0.05; ** *P* = 0.001 to <0.01; ***P < 0.001; all *unmarked correlations* are not significant (*P* > 0.05)

The analysis was repeated by restricting the data to virus-positive samples (or wells) to minimize the effect of non-detects on the correlations. With this restricted data set, the highest correlations to culturable virus were *E. coli* (rho = 0.62, *P* < 0.001, *n* = 144) and somatic coliphage (rho = 0.54, *P* < 0.001, *n* = 141) on a per-sample basis and somatic coliphage (rho = 0.48, *P* < 0.001, *n* = 76) on a per-well basis; however, restricting the data set did not improve the correlations among the indicators and virus detected by molecular assays (data not shown).

Subsequently, the association between indicator and virus occurrence was evaluated (detect or non-detect) using logistic regression models and the data from the 12 studies. Culturable viruses were associated at statistically significant levels with the indicators except for total coliforms measured at the sample level and spores measured at the well level (Table 8). In contrast, PCR-viruses and indicators at the sample level were never statistically associated, and at the level of well only three of the six indicators (total coliforms, *E. coli*, and somatic phage), were significantly associated with PCR-viruses. The two most commonly used indicators in the US—total coliforms and *E. coli*—were associated at the well level with both culturable viruses and PCR-viruses, albeit the *E. coli* and culturable virus association was marginally not significant (*P* = 0.087; Table 8).

**Table 8:** Indicator and virus associations from the 12 groundwater virus studies^a^

Indicator	Viruses measured by culture				Viruses measured by PCR			
	Sample-level analyses		Well-level analyses		Sample-level analyses		Well-level analyses	
	Risk ratio^b^	*P* ^c^	Risk ratio	*P*	Risk ratio	*P*	Risk ratio	*P*
Total coliform	2.6	0.164	4.5	0.043	1.0	0.881	1.3	0.037
*E. coli*	7.0	<0.001	3.1	0.087	0.9	0.795	1.6	0.008
Enterococcus	5.7	<0.001	4.5	0.002	1.0	0.858	1.0	0.913
F-specific phage	4.4	0.036	7.7	0.037	1.2	0.297	1.2	0.393
Somatic phage	9.0	<0.001	9.1	<0.001	1.7	0.176	1.9	<0.001
Spores	3.3	<0.001	1.5	0.549	0.8	0.592	1.4	0.342

^a^Associations were evaluated at the level of sample (i.e., comparison between indicator and virus samples collected at the same time from a well) and at the level of well (i.e., designating a well as positive for an indicator or virus based on one or more positive results from multiple samples collected from that well). All analyses were adjusted for the effects of study and well (sample-level analyses) or the effect of study (well-level analyses)

^b^ *Risk ratio* = positive predictive value/(1-negative predictive value)

^c^ *P* is for the overall indicator-virus association

The risk ratios reported in Table 8 give the relative increase in the probability of detecting a virus-positive sample (or well) when an indicator is detected compared to when an indicator is not detected—for example, a positive *E. coli* sample is associated with a seven-times greater chance of detecting culturable viruses in a corresponding sample compared to a negative *E. coli* result. For PCR-viruses, the chance of detection in a sample is similar whether a corresponding *E. coli* sample is positive or negative (risk ratio = 0.9, *P* = 0.79). However, at the level of well, an *E. coli* positive well is associated with a 60% greater chance the well, at some time, will be positive for PCR-viruses (risk ratio = 1.6, *P* = 0.008).

Table 8 reports the associations and risk ratios adjusted for study (i.e., accounting for underlying differences among the 12 studies) and the sample-level analyses additionally include an adjustment for wells. Analyses were also conducted without these adjustments (data not shown). Generally, the adjustments resulted in lower estimated risk ratios and similar conclusions regarding statistical significance of the indicator-virus association, suggesting the probability of detecting a virus when an indicator is present differed across the individual studies and the wells within each study. The effect of the adjustments on the risk ratios could reflect differences in hydrogeological settings, virus contamination sources, laboratory methods, or several unknown factors at the level of study or well that are related to virus and indicator occurrence. Nonetheless, for this evaluation of indicators and viruses in groundwater, it is evident it was important to account for the effects of both study and well.

Indicator test performance measures were examined next. Sensitivities of the indicators for signifying whether a virus was detected in a sample or well were low (2–30%), with estimates being relatively higher when virus positivity was determined by culture (11–30%, Table 9) versus PCR (2–12%, Table 10); note that all test performance measures reported here are adjusted for study and well (sample-level analyses) or study only (well-level analyses). By culture, between 11 and 30% of virus-positive samples and between 37 and 73% of virus-positive wells could be correctly identified as virus-positive by an indicator. In other words, many samples and wells negative for an indicator were, in fact, positive for virus. The corresponding numbers for PCR were ≤12% for samples and ≤39% for wells.

**Table 9:** Indicator test performance for indicating the presence of human viruses measured by culture^a^

Indicator	Sample-level analyses				Well-level analyses			
	Sensitivity (%)^b^	Specificity (%)^c^	Positive predictive value (%)^d^	Negative predictive value (%)^e^	Sensitivity (%)	Specificity (%)	Positive predictive value (%)	Negative predictive value (%)
Total Coliform	29 (8–65)	79 (51–93)	7 (2–25)	97 (88–99)	73 (28–95)	64 (32–87)	23 (7–57)	95 (79–99)
*E. coli*	26 (14–44)	95 (90–98)	18 (5–48)	97 (90–99)	37 (15–66)	88 (69–96)	29 (7–70)	91 (65–98)
Enterococcus	30 (13–55)	92 (85–96)	11 (5–23)	98 (96–99)	57 (23–85)	80 (59–92)	24 (8–54)	95 (80–99)
F-specific phage	11 (3–35)	97 (92–99)	10 (2–39)	98 (95–99)	50 (14–86)	92 (59–99)	40 (6–86)	95 (80–99)
Somatic phage	14 (3–45)	97 (90–99)	16 (6–36)	98 (96–99)	49 (17–82)	94 (78–99)	46 (14–81)	95 (82–99)
Spores	19 (2–74)	95 (62–100)	9 (4–18)	97 (96–98)	48 (6–93)	71 (42–89)	32 (17–53)	79 (46–94)

^a^All sample and well results from the combined 12-study dataset were included in the analysis. Performance was evaluated at the level of sample (i.e., comparison between indicator and culturable virus samples collected at the same time from a well) and at the level of well (i.e., designating a well as positive for an indicator or virus based on one or more positive results from multiple samples collected from that well). Analyses were adjusted for the effects of study and well (sample-level analyses) or the effect of study (well-level analyses). *Values in parentheses* are the 95% confidence intervals

^b^ *Sensitivity* = the percentage of virus-positive samples or wells the indicator correctly identified as virus-positive

^c^ *Specificity* = the percentage of virus-negative samples or wells the indicator correctly identified as virus-negative

^d^ *Positive predictive value* = the percentage of indicator-positive samples or wells that were virus positive

^e^ *Negative predictive value* = the percentage of indicator-negative samples or wells that were virus negative

**Table 10:** Indicator test performance for indicating the presence of human viruses measured by molecular methods^a^

Indicator	Sample-level analyses				Well-level analyses			
	Sensitivity (%)	Specificity (%)	Positive predictive value (%)	Negative predictive value (%)	Sensitivity (%)	Specificity (%)	Positive predictive value (%)	Negative predictive value (%)
Total Coliform	12 (3–35)	88 (68–96)	9 (4–20)	91 (82–96)	33 (14–58)	75 (47–91)	33 (13–62)	75 (46–92)
*E. coli*	2 (1–10)	97 (91–99)	9 (3–21)	91 (82–96)	12 (5–29)	94 (83–98)	40 (15–71)	74 (45–91)
Enterococcus	4 (1–12)	96 (88–98)	7 (2–19)	93 (83–97)	14 (3–52)	85 (50–97)	26 (5–69)	73 (32–94)
F-specific phage	5 (2–12)	96 (90–98)	9 (3–22)	93 (83–97)	17 (5–44)	87 (68–96)	31 (7–74)	75 (38–94)
Somatic phage	4 (1–18)	98 (89–100)	12 (3–36)	93 (82–98)	15 (5–39)	94 (82–98)	45 (14–81)	76 (40–94)
Spores	5 (1–31)	94 (60–100)	6 (1–42)	92 (69–98)	39 (8–83)	74 (41–92)	69 (15–97)	50 (4–96)

^a^See footnotes to Table 9 for a description of procedures and test performance measures

Positive predictive values were also low for the indicators in predicting the detection of both culturable virus and PCR-virus. Many samples and wells that were positive for an indicator were, in fact, negative for virus (i.e., the indicators had a high rate of false positives). Positive predictive values at the well level were higher (but still mostly <50%) than at the sample level. Approximately 30 to 40% of the wells that were positive for an indicator were contaminated with virus at some point during the sampling period.

In contrast, specificities of the indicators were high, with values often near 90% or greater for culturable virus and PCR-virus. For example, *E. coli* had 95 and 97% specificity at the sample level for culturable virus and PCR-virus, respectively. In other words, a negative *E. coli* result correctly identified 95–97% of the samples that were negative for viruses. Specificity values at the well level were consistently lower than at the sample level for all six indicators, but the magnitude of the differences were usually not large.

Negative predictive values for the indicators were generally greater than 90%, with the exception of PCR-viruses at the well level, where estimates ranged from 50 to 76%. An indicator with a high negative predictive value means that when a sample or well is indicator-negative, it is likely to be also virus-negative (i.e. the indicator has a low rate of false negatives).

The sample and well data were reanalyzed according to the TCR, Hydrogeology, and GWR susceptibility subsets described in Table 5. Table 11 shows the ratio of the percent of virus-positive samples or wells in each subset to the percent of all virus positive samples or wells. Culturable viruses were twice as likely to be present in samples obtained from wells in each subset as in samples from all wells. Viruses measured this way were only 1.3–1.5 times more likely in wells from the TCR and hydrogeology subsets, but 4 times more likely in the GWR subset. PCR virus was poorly associated with the different subsets (ratios of 0.9–1.6).

**Table 11:** Ratio of % positive in category/overall % positive^a^

Susceptibility category	Samples		Wells	
	Culturable virus	PCR-virus	Culturable virus	PCR-virus
TCR	1.9 (417)^b^	1.1 (672)	1.3 (131)	1.2 (148)
Hydrogeology	1.9 (407)	0.7 (333)	1.5 (131)	0.9 (65)
GWR	2.2 (477)	1.1 (599)	3.9 (59)	1.6 (118)

^a^See Table 5 for category descriptions

^b^ *Values in parentheses* are the number of samples or wells in the category

Table 12 shows the indicator test performance measures for those combinations of indicators and susceptibility categories that had statistically significant indicator-virus associations. The chance of detecting virus was elevated when total coliform or enterococci bacteria or F-specific bacteriophage were detected in a hydrogeological susceptibility setting and when enterococci or somatic bacteriophage were detected for wells considered susceptible by having a TCR violation. No single indicator improved the chance of detecting virus for those wells considered susceptible by the GWR. Compared to the analyses that included all samples or wells, sensitivities and positive predictive values generally were higher for indicators measured in wells in all three susceptibility categories for both culturable virus and PCR-virus.

**Table 12:** Indicator test performance measures and risk ratios for wells placed into susceptibility categories^a^

Indicator	Analysis basis	Category^b^	Sensitivity	Specificity	Positive predictive value	Negative predictive value	Risk ratio	*P*
*Culturable virus*								
Total coliforms	Samples	All	29	79	7	97	2.6	0.164
		Hydrogeology	58	66	14	96	3.8	0.016
	Wells	All	73	64	23	95	4.5	0.043
		Hydrogeology	88	55	42	93	6.2	0.016
Enterococci	Wells	All	57	80	24	95	4.5	0.002
		Hydrogeology	73	77	41	93	5.8	0.01
		TCR	82	39	36	93	5.1	0.022
F-specific phage	Samples	All	11	97	10	98	4.4	0.036
		Hydrogeology	15	97	27	96	7.2	0.004
	Wells	All	50	92	40	95	7.7	0.037
		Hydrogeology	58	96	76	91	8.4	0.005
*PCR-virus*								
Enterococci	Wells	All	14	85	26	73	1.0	0.913
		TCR	88	38	43	91	4.9	0.014
F-specific phage	Samples	All	5	96	9	93	1.2	0.297
		Hydrogeology	5	97	10	95	2.2	0.019
Somatic phage	Wells	All	15	94	45	76	1.9	<0.001
		TCR	42	86	56	80	2.8	0.043

^a^Data are reported only for susceptibility categories (see Table 5) and indicators that have statistically significant indicator-virus associations. All values were adjusted for study (i.e., accounting for underlying differences among the 12 studies) and the sample-level analyses additionally include an adjustment for well

^b^The *All* category data are from Tables 9 and 10 and are included here for easy comparison with the susceptibility categories

## Discussion

This study addresses the relationship between microbial indicators and human enteric virus in groundwater. The focus of the study is on public systems in the US, Canada, Europe, and Asia for which raw data from 746 wells and 2273 samples were available. One or more indicator was found in 44% of the wells, while culturable virus was detected in 8% and PCR virus in 30% of the wells.

Many studies have examined correlations between concentrations of indicators and viruses and often times the correlations are weak or non-existent (see references in Table 1). When 12 studies were combined, the correlations between indicator and virus concentrations were statistically significant, but the low rho values showed the correlations were weak. Contributing factors to the weak relationships are the use of different methodologies, the amalgamation of wells from various hydrogeological settings, and the large number of samples or wells in the dataset that were negative for indicators or viruses. Excluding most negative values from the Spearman rank order test did increase the rho values pertaining to culturable virus, but as negative values cannot be excluded a priori such a relationship has no practical value for well testing.

Hynds et al. (2014) pooled data from 39 studies of private groundwater wells that included both microbial indicator and pathogen data and compared the percentage of indicator positive wells to the percentage of pathogen positive wells. The correlations tended to be weak and some were opposite of what would be expected, for example, viruses in groundwater were negatively correlated with enterococci. These authors recommend direct testing for virus. From 540 sets of indicator-pathogen correlations in studies of surface water, Wu et al. (2011) modeled the probability of finding a significant correlation and concluded that many studies do not find a correlation because of small sample size. Payment and Locas (2011) compared virus concentrations to concentrations of six indicators in 242 samples from 25 groundwater sites and found no correlations.

Logistic regression was used as an alternative approach to examine the association between dichotomous representations of indicator and virus detections. This approach allowed the calculation of risk ratios (i.e. the ratio of virus detection probabilities in the presence versus the absence of an indicator) while adjusting the association for underlying differences among the 12 studies and among wells within a study. Risk ratios and tests for association evaluate the overall correspondence between indicator and virus occurrence. A more complete interpretation of an indicator’s value with respect to viruses is provided by calculating standard test performance measures (e.g., sensitivity, specificity, etc.) that convey the likelihood of false positives and false negatives (Borchardt et al. 2003).

The risk ratios show that, in general, culturable viruses are more likely to be detected when there is a positive indicator result, especially for the indicators *E. coli*, enterococci and somatic phage at the sample level and F-specific and somatic phage at the well level. For example, the data indicate that it is nine times more likely to find culturable virus in a sample or well that also has somatic coliphage. In contrast, no significant risk ratios were found among indicators and PCR-virus at the sample level and the strongest risk ratios at the well level were less than two. Similarly, in examining the factors associated with statistically significant correlations between indicators and pathogens in surface waters, Wu et al. (2011) showed that studies that use molecular methods for pathogen detection were less likely to find significant correlations.

All six indicators that were evaluated tended to have low sensitivity and low positive predictive values, but high specificity and high negative predictive values. From a practical standpoint, this means that if a well has an unknown virus contamination problem, it is unlikely to be identified by a positive indicator (low sensitivity). Additionally, a positive indicator result does not necessarily mean the well is virus-contaminated; it could likely be a false positive (low positive predictive value). If the well does not have a virus contamination problem, there is a reasonable chance the indicator results will be negative, confirming there is no problem (high specificity). And a well that is indicator negative is unlikely to be virus contaminated, especially for culturable viruses (high negative predictive value). In summary, the downside of the indicators is that many virus positive wells can be missed and there could be many false positives. On the other hand, wells that are indicator-negative are unlikely to have virus contamination problems. Moreover, sensitivities and positive predictive values at the well level were higher for all six indicators compared to the sample level, for the most part without sacrificing a large drop in specificity or negative predictive value. This suggests that multiple samples from a well improves indicator performance in assessing a well’s susceptibility to virus contamination. Indicator performance might also be improved by considering not just the detection of the indicator, but also its concentration. Payment and Locas (2011) showed the probability of human virus detection in the Saint-Lawrence River in Quebec, Canada, increased with increasing concentrations of the indicators thermotolerant coliforms or *Clostridia perfringens*. An indicator’s sensitivity performance measure may depend on the indicator concentration with higher sensitivities at higher concentrations.

The indicator-virus associations were evaluated for three well susceptibility categories to examine if indicators were more informative based on prior knowledge of well-specific data (Table 5). The TCR category was chosen because total coliforms are the most common indicator used to evaluate well susceptibility to pathogens. The hydrogeology category was selected due to the known susceptibility of karst, fractured bedrock, and gravel/cobble settings, and the GWR category was selected because the criteria for well susceptibility includes two indicators that typically had not been tested in groundwater, enterococci and coliphage. Among the wells in the three susceptibility categories, culturable virus was detected twice as frequently in samples and 1.3–3.9 times more in wells compared to all samples or wells (Table 11). In contrast, PCR-viruses were detected only 0.7–1.6 times more in samples or wells in the susceptibility categories. This difference may be a function of virus fate in the subsurface. It may be easier to detect viruses by PCR in less susceptible settings as the viruses can still be detected long after infectivity is lost and at greater distances from sources of contamination (Ogorzaly et al. 2010).

Sensitivities, positive predictive values, and risk ratios tended to be higher for total coliforms, enterococci, F-specific phage, and somatic phage when they were measured in samples or wells in the TCR or hydrogeology susceptibility categories. In other words, for wells with two or more TCR violations or wells located in susceptible hydrogeological settings, like karst, these indicators performed better at identifying samples and wells that were true virus-positives and with a lower false positive rate. However, for wells that would have met the criteria under the GWR for additional monitoring, none of the single indicators performed any better than when they were measured in all wells regardless of susceptibility status.

In interpreting the data presented in the preceding, it needs to be stressed that there is a degree of uncertainty in the placement of wells into the susceptibility categories, particularly the hydrogeology category. Interpretation is weakened by the lack of uniformity in the types of stressors measured by individual studies as well as by the different methodologies used. Future studies that examine indicator and virus relationships should measure viruses by both culture and molecular assays, and include at least total coliform, *E. coli*, enterococci, aerobic spores, F-specific and somatic coliphages. Standard methods should be used as much as possible.

The finding of a virus by either culturable or molecular assays is not always easily translatable to the public health risk presented by a particular well. Virus concentration in groundwater can vary rapidly over time (Bradbury et al. 2013). A single data point can fall anywhere within the possible range of virus concentrations during transient contamination, from having no virus, and thus underestimating risk, to by chance collecting the sample during peak concentration and overestimating risk. In addition, as virus recovery from water by various concentration procedures rarely achieves 100% (Cashdollar and Wymer 2013; Gibbons et al. 2010; Ikner et al. 2012, 2011; Karim et al. 2009; Sobsey and Glass 1984; Wu et al. 2013) virus concentration, and therefore risk, can be underestimated. Different virus groups and even different members of the same species can have different recovery efficiencies, while recovery efficiency of a single virus type will vary over time with changes in turbidity and other water quality factors.

To pose a health risk, virus concentrations in the well water must be high enough that individuals drinking the water ingest more virus than the minimum infectious dose necessary to initiate infection. Apart from egregious virus contamination of groundwater that results in an outbreak (e.g. Borchardt et al. 2011), most surveys of groundwater in developed countries show culturable virus concentrations that typically are very low (e.g., combined data from this study), suggesting the health risk is low. On the other hand, culturable virus assays can underestimate virus concentrations and occurrence. Virus in water can be aggregated with each aggregate registering as a single infectious unit in culturable virus assays (Gassilloud and Gantzer 2005); however, aggregates may have a sufficient number of virus particles to cause an infection, if consumed. Culturable assays also underestimate risk in that they do not detect many important waterborne enteric viruses, and cell culture does not necessarily replicate the same favorable conditions for virus replication as when a virus infects a host.

Because PCR detects virus genomes and not necessarily infectious virions, PCR measurements of viruses in groundwater are often thought to overestimate the health risk. On the other hand, virus present can maintain infectivity for months at temperature of 12 °C or less (Charles et al. 2009; Nasser and Oman 1999). PCR also may underestimate risk because enteric viruses, and especially RNA viruses, evolve constantly, leading to genetic variability that prevents their detection by PCR (i.e., false negative results). Nonetheless, the presence of viral genomes in groundwater shows that an aquifer is susceptible to fecal contamination. Importantly, Borchardt et al. (2012) compared the fraction of acute gastrointestinal illness (AGI) associated with drinking untreated groundwater in 14 communities with PCR-virus from community wells. The concentration of enteroviruses in the tap water of households in the communities was associated with AGI in adults, and tap water concentrations of norovirus genogroup I were associated with AGI in all ages. Using quantitative microbial risk assessment, the authors estimate that between 6 and 22% of the overall AGI in the communities and up to 63% of AGI in children less than 5 years of age was associated with drinking untreated tap water. The AGI incidence increased by 63% when the mean norovirus concentration in the communities’ tap water increased from 0 to about 3 genomic copies per liter. Thus, without results to the contrary, the possibility of adverse health risks based upon PCR positive findings should be considered when there are septic tanks, sewer lines, or other sources of contamination near aquifers that reside in similar hydrogeologic settings to those in Wisconsin.

## Conclusions

A meta-analysis of 12 groundwater studies was conducted. Overall, one or more indicators were present in 37% of samples and 44% of wells, culturable virus in 4% of samples and 8% of wells, and PCR-virus in 14% of samples and 30% of wells. All six indicators examined in this study were associated with culturable virus at one or both levels of analysis (sample or well). None of the indicators were associated with PCR-measured viruses at the sample level, and only total coliforms, *E. coli*, and somatic phage were associated with PCR-viruses at the well level. Judging by the risk ratios, the best indicator for culturable virus and PCR-virus in groundwater was somatic phage. However, all the indicators tended to have low sensitivities and positive predictive values, but high specificity and negative predictive values, which means groundwater that tests negative for the indicators is unlikely to be virus contaminated, but positive indicator tests do not necessarily mean there is virus contamination. That the sensitivities and positive predictive values of the indicators were higher at the well-level of analysis matches intuition: evaluating well susceptibility to virus contamination is improved by sampling for indicators multiple times from the well. In combining the 12 studies, it was learned that the statistical models had to account for underlying differences among the studies and wells, and furthermore, the indicator-virus associations changed when the analysis was restricted to wells classified as susceptible by the total coliform rule or hydrogeology. This suggests the strength of associations between indicators and viruses are specific to hydrogeological setting. Thus, for optimal management of groundwater sanitary quality, the indicator associations and test performance measures, as reported in this study, should be characterized for the specific region under management. This would give groundwater managers the greatest confidence in interpreting indicator test results for their region. This study focused upon the relationships of single microbial indicators to virus occurrence in groundwater samples and wells. Future studies should examine whether multiple microbial indicators would provide stronger correlation to virus presence.
